# Рак щитовидной железы у ребенка с синдромом Коудена

**DOI:** 10.14341/probl13445

**Published:** 2024-11-04

**Authors:** Э. Б. Бричева, Е. В. Нагаева, Д. Н. Бровин, Е. В. Бондаренко, М. С. Шеремета, О. Б. Безлепкина, Т. С. Олина, Т. В. Коваленко

**Affiliations:** Национальный медицинский исследовательский центр эндокринологии; Национальный медицинский исследовательский центр эндокринологии; Национальный медицинский исследовательский центр эндокринологии; Национальный медицинский исследовательский центр эндокринологии; Национальный медицинский исследовательский центр эндокринологии; Национальный медицинский исследовательский центр эндокринологии; Республиканская детская клиническая больница; Ижевская государственная медицинская академия

**Keywords:** синдром Коудена, PTEN, синдром множественных гамартом, рак щитовидной железы, дети

## Abstract

Болезнь Коудена (синдром Коудена) относится к PTEN-ассоциированным синдромам гамартомных опухолей. Возникает в связи с мутацией в гене гомолога фосфатазы и тензина, одной из основных функций которого является регуляция клеточного цикла. Наличие мутации в гене приводит к неконтролируемому росту клеток, а пациенты имеют пожизненный повышенный риск возникновения новообразований различной степени злокачественности. В статье представлен клинический случай синдрома Коудена с ранним дебютом — в 7 лет. Патогномоничным для синдрома Коудена является сочетание макроцефалии (SDS окружности головы >2) с различными кожными проявлениями (трихилеммомы лица, акральный кератоз, папилломатозные папулы), наличие доброкачественных и/или злокачественных образований. Из злокачественных образований чаще всего встречаются рак молочной и щитовидной желез, колоректальный рак, почечно-клеточная карцинома и рак эндометрия. Доказано, что карцинома щитовидной железы имеет более ранний возраст манифестации и зачастую встречается уже в детском возрасте. Это определяет необходимость скрининга пациентов с доказанной мутацией в гене PTEN на наличие узловых образований с раннего возраста. При необходимости выполнения хирургического лечения предпочтительным остается тиреоидэктомия в связи с частыми рецидивами узлообразования, а также неопределенным потенциалом злокачественности ввиду малой изученности узлов щитовидной железы у пациентов с мутациями в гене PTEN.

## Abstract (en)

Cowden disease (Cowden syndrome) refers to PTEN-associated hamartoma tumor syndromes. It arises due to a mutation in the phosphatase and tensin homolog gene, one of the main functions of which is cell cycle regulation. The presence of a mutation in the gene leads to uncontrolled cell growth, and patients have a lifelong increased risk of neoplasms of various degrees of malignancy. This article presents a clinical case of Cowden syndrome with an early debut at the age of 7 years. The combination of macrocephaly (SDS of head circumference >2) with various skin manifestations (facial trichilemmomas, acral keratosis, papillomatous papules) and the presence of benign and/or malignant neoplasms are pathognomonic for Cowden syndrome. Of the malignancies, breast and thyroid cancer, colorectal cancer, renal cell carcinoma, and endometrial cancer are the most common. Thyroid carcinoma has been shown to have an earlier age of manifestation and often occurs already in childhood. This determines the need to screen patients with a proven mutation in the PTEN gene for nodal neoplasms from an early age. If surgical treatment is necessary, thyroidectomy remains preferable due to the frequent recurrence of nodules, as well as the uncertain potential for malignancy due to the low study of thyroid nodules in patients with mutations in the PTEN gene.

## АКТУАЛЬНОСТЬ

Синдром множественных гамартом (опухолевый синдром гамартом) — группа наследственных заболеваний с аутосомно-доминантным типом передачи, обусловленная мутацией в гене PTEN [1]. К ним относятся синдром Коудена, синдром Баннаяна-Райли-Рувалькаба, Протей-подобный синдром, синдром Лермитта-Дюкло и ювенильный младенческий полипоз [2].

Ген гомолога фосфатазы и тензина (PTEN — Phosphatase and tensin homolog), расположенный на участке хромосомы 10q23, представляет собой ген-супрессор опухолевого роста, играющий важную роль в регуляции клеточного цикла. Мутации в гене PTEN приводят к потере контроля над скоростью деления клеток, обусловливая тем самым их быстрый и неконтролируемый рост [1][3].

Наиболее распространенное проявление мутации — синдром Коудена — имеет очень редкую встречаемость в популяции: 1 случай на 200 000–250 000 человек [4]. Классическими признаками синдрома Коудена являются макроцефалия, множественные поражения кожи и слизистых оболочек, а также гамартомы различных органов. Помимо этого, пациенты с синдромом Коудена имеют высокий пожизненный риск развития злокачественных новообразований, среди которых чаще всего встречаются рак молочной и щитовидной желез, эндометрия, почек и кишечника [5-7]. При этом только дифференцированный рак щитовидной железы (ДРЩЖ) имеет высокий риск развития не только у взрослых (35–38%), но и у детей (4–12%) с синдромом Коудена, в то время как риск возникновения других видов рака у детей равен общепопуляционному [8]. Описано, что не все узловые образования щитовидной железы (ЩЖ) первоначально носят злокачественный характер, до 50% из них являются доброкачественными [8]. Вместе с тем до сих пор нет точных данных о потенциале таких узловых образований, в связи с чем наиболее предпочтительным методом лечения остается тиреоидэктомия [8-10]. Все это обусловливает необходимость проведения молекулярно-генетического тестирования при отягощенной наследственности и обязательного скрининга на наличие узловых образований в ЩЖ у детей с уже установленной мутацией в гене PTEN.

В статье представлен клинический случай ранней манифестации синдрома Коудена, описывающий появление герминогенной опухоли яичника и папиллярного рака щитовидной железы у ребенка.

## ОПИСАНИЕ СЛУЧАЯ

В ноябре 2019 г. в ФГБУ «НМИЦ эндокринологии» впервые была госпитализирована девочка в возрасте 10 лет 7 мес. с жалобами на наличие узловых образований в обеих долях ЩЖ. Наследственный анамнез отягощен по онкологическим заболеваниям со стороны матери: двоюродный дедушка — саркома внутренних органов, смерть в 36 лет; двоюродная бабушка — рак молочной железы, смерть в 50 лет; у другой двоюродной бабушки (на момент написания статьи — 70 лет, жива) в анамнезе — тиреоидэктомия по поводу РЩЖ, родной дядя умер в возрасте 45 лет, причина внезапной смерти не установлена (рис. 1).

**Figure:**
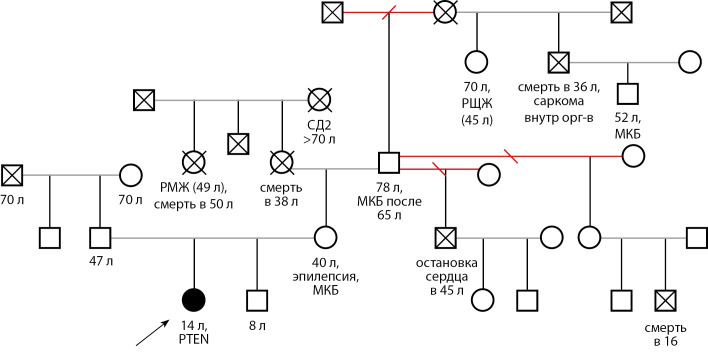
Рисунок 1. Генеалогическое древо семьи Т.

Из анамнеза известно, что в ноябре 2016 г. (в возрасте 7 лет 7 мес) в связи с жалобами на боли в правой подвздошной области ребенок был госпитализирован с предварительным диагнозом «острый аппендицит». В ходе обследования обнаружен крайне высокий уровень альфа-фетопротеина (АФП) — 925 МЕ/мл, на мультиспиральной компьютерной томографии (МСКТ) — признаки объемного кистозно-солидного образования правого яичника с четкими неровными контурами размерами 133х69х150 мм, равномерно накапливающее контрастное вещество. Образование распространялось на нижнюю треть брюшной полости, смещая прилегающие органы (преимущественно петли кишечника) без убедительных данных за врастание. Выявлены парааортальные, бифуркационные узлы до 8 мм в поперечнике. Выполнено удаление правого яичника с придатками. По результатам иммуногистохимического исследования описана картина смешанной герминогенной опухоли, включающая 90% зрелой тератомы с дермоидной, респираторной и мезенхимальной дифференцировкой и 10% опухоли желточного мешка (G2-3). После операции девочке была проведена полихимиотерапия (ПХТ): 3 блока ВЕР (блеомицин, этопозид, цисплатин) с положительным результатом.

Через месяц, в декабре 2016 г., во время госпитализации для проведения ПХТ при ультразвуковом исследовании (УЗИ) ЩЖ обнаружено узловое образование в левой доле. В течение 2 лет динамического наблюдения (декабрь 2016 — февраль 2019 гг.) зафиксировано выраженное увеличение количества и размеров узловых образований. При УЗИ ЩЖ от февраля 2019 г.: в правой доле — множественные участки изоэхогенной структуры; в левой — 2 узла размерами 16х22х16 мм и 5х4х6 мм (EU-TIRADS 3), по результатам тонкоигольной аспирационной биопсии (ТАБ) — Bethesda II.

В ноябре 2019 г. (в 10 лет 7 мес) девочка впервые поступила в ФГБУ «НМИЦ эндокринологии». При объективном осмотре: антропометрические показатели соответствовали хронологическому возрасту (рост — 138 см, SDS роста: -0,38, вес — 37,6 кг, ИМТ — 19,7 кг/м2, SDS ИМТ: 0,93), окружность головы — 59 см (SDS: 4,24). Кожные покровы чистые, без патологических высыпаний. Щитовидная железа расположена типично, мягко-эластической консистенции, пальпировалось округлое узловое образование диаметром около 2 см в левой доле ЩЖ, шейные лимфатические узлы увеличены с двух сторон. Половые органы сформированы правильно, по женскому типу, Таннер 3, Ме отсутствовали. По данным лабораторной диагностики: гормональные и биохимические показатели крови без клинически значимых изменений. При УЗИ ЩЖ: в правой доле визуализировались 3 анэхогенных участка диаметром до 0,4 см с четкими контурами (EU-TIRADS 2-3), в левой доле — 3 узловых образования: в верхней трети доли — изоэхогенный узел с четкими контурами 2,3х1,5х1,3 см (EU-TIRADS 2-3), в нижней трети — два прилежащих друг к другу образования с четкими контурами, умеренно пониженной эхогенности 0,7х0,6х0,5 см и 0,9х0,7х0,6 см (EU-TIRADS 4). Проведена ТАБ узлов левой доли, по данным цитологического заключения: фолликулярная неоплазия (Bethesda IV). В феврале 2020 г. (в возрасте 10 лет 11 мес) выполнена гемитиреоидэктомия слева. По результатам морфологического исследования выявлена фолликулярная аденома левой доли ЩЖ, рекомендовано динамическое наблюдение.

Через год, в марте 2021 г., в возрасте 12 лет, при повторном обследовании в ФГБУ «НМИЦ эндокринологии» был зафиксирован рецидив двустороннего многоузлового зоба. При УЗИ ЩЖ: общий объем железы — 4,5 см3; правая доля — 4,0х1,5х1,3 см, объем — 3,7 см3; остаточная ткань левой доли: 2,5х1,0х0,7 см, объем 0,8 см3. В обеих долях определялись образования с четкими контурами гипо- и изоэхогенной структуры диаметром до 0,7 мм. Учитывая малый размер узлов, их доброкачественные характеристики, было принято решение продолжить динамическое наблюдение с контролем УЗИ ЩЖ через 6 месяцев.

В связи с отягощенным семейным анамнезом, сочетанием новообразований яичника и щитовидной железы, было проведено молекулярно-генетическое исследование — наиболее вероятным представлялось присутствие патогенного варианта в гене DICER-1, однако патогенный гетерозиготный вариант c.380G>A:p.G127E был обнаружен в 5 экзоне гена PTEN. Данный вариант ранее в литературе не описан, но другая аминокислотная замена в 127 кодоне PTEN (G127R, HGMD:CM128490) ранее описана при болезни Коудена.

При госпитализации в сентябре 2021 г. (в возрасте 12 лет 5 мес), учитывая отрицательную динамику данных УЗИ ЩЖ, повторно была проведена ТАБ узловых образований обеих долей: правая доля — Bethesda-II, левая доля-Bethesda-IV. В связи с выраженным рецидивирующим характером узлового зоба, ростом образований и изменениями их сонографических и цитологических характеристик, а также наличием подтвержденной патогенной мутации в гене PTEN, в октябре 2021 г. (в возрасте 12 лет 6 мес) была проведена окончательная тиреоидэктомия, включающая удаление правой доли, а также перешейка с остаточной тканью левой доли ЩЖ. В ходе оперативного вмешательства был визуализирован и удален подозрительный паратрахеальный лимфоузел. По результатам морфологического исследования выявлен папиллярный рак ЩЖ солидно-фолликулярного строения с мультифокальным типом роста пораженной правой доли и перешейка размером до 0,6 см (pT1a(m)NxMx. В послеоперационном периоде у ребенка развилась клиническая картина гипопаратиреоза, в связи с чем, помимо левотироксина натрия, была инициирована заместительная терапия препаратами кальция и активными формами витамина Д.

В марте 2022 г., в возрасте 13 лет, девочке была проведена плановая радиойодтерапия 131I активностью 2,65 Гбк. На фоне 3-недельной отмены левотироксина натрия в гормональном профиле отмечался определяемый низкий уровень тиреоглобулина (ТГ) — 2 нг/мл (3,5–77), АТ к ТГ — 10 МЕ/мл (0–115). При УЗИ ложа: ЩЖ визуализировались множественные лимфоузлы неоднородной структуры с гиперэхогенными включениями: справа — до 1,0 см в диаметре, слева — до 1,9 см. По результатам посттерапевтической сцинтиграфии в режиме «все тело», описаны признаки накопления 131I в проекции ложа ЩЖ (3,1%), что соответствует остаточной тиреоидной ткани, патологического включения радиофармпрепарата (РФП) в легких и скелете не выявлено.

Через полтора года после радиойодтерапии выполнена диагностическая сцинтиграфия с введением 131I активностью 70 МБк в режиме «все тело»: патологического накопления РФП не обнаружено. В лабораторных показателях зафиксирован неопределяемый уровень ТГ — 0,04 нг/мл (3,5–77), низкий уровень АТ к ТГ — 10 МЕ/мл (0–115), на фоне высокого уровня ТТГ — 91,3 мМЕ/л. По данным УЗИ ложа ЩЖ, данных за рецидив новообразования не обнаружено. Установлена полная лабораторная и инструментальная ремиссия заболевания.

## ОБСУЖДЕНИЕ

В 1963 г. K.M. Lloyd et al. впервые описали клинический случай мультисистемного заболевания, включающего в себя папилломатоз губ и ротовой полости, многоузловой зоб ЩЖ и рак молочной железы у пациентки с фамилией Коуден [11]. С тех пор было описано много клинических случаев болезни Коудена, что, несомненно, помогло определить патогномоничные фенотипические проявления, а также установить механизм их возникновения.

Ген гомолога фосфатазы и тензина кодирует белок, который принимает непосредственное участие в регуляции клеточного цикла. Продукт гена PTEN напрямую подавляет один из наиболее важных механизмов развития рака: сигнальный путь фосфатидилинозитол-3-киназы (PI3K), способствуя снижению клеточной пролиферации и стимулируя явление апоптоза в клетке. Гетерозиготные мутации в гене PTEN приводят к потере его главной функции — супрессии избыточного роста клеток. Потеря активности PTEN вызывает усиление фосфорилирования различных клеточных белков, оказывая влияние на рост, миграцию и апоптоз клеток. Чрезмерная активация пути PI3K/AKT/mTOR вследствие дефицита PTEN может привести к избыточному росту клеток, увеличивая тем самым риски развития новообразований различной степени злокачественности в течение всей жизни [1][3][12].

Множество исследований доказывают, что заболевания, входящие в группу PTEN-ассоциированных синдромов, не имеют четкой корреляционной зависимости между генотипом и фенотипом [1][13][14]. Подтверждением этому служит мультисистемный характер поражения органов с отсутствием четких критериев, разграничивающих синдромы друг от друга. В части исследований главным образом подчеркивается возрастная зависимость между синдромами Коудена и Баннаяна-Райли-Рувалькаба [14][15][16]. В зарубежной литературе есть описание случаев возникновения двух этих синдромов у кровных родственников при наличии одной и той же патогенной мутации в гене PTEN [15].

Несмотря на большое количество характерных компонентов, сохраняются трудности в диагностике PTEN-ассоциированных синдромов. В настоящее время Национальной комплексной онкологической сетью (NCCN — National Comprehensive Cancer Network) официально утверждены критерии диагностики только для синдрома Коудена [17] (табл. 1). В основу их легли характеристики, сформулированные еще в 1996 г. Международным консорциумом Коудена, а также данные системы клинической оценки, разработанной на основании проспектового исследования 3042 пробандов с синдромом Коудена, описанного Tan et al. [18][19]. Согласно критериям, диагноз синдрома Коудена требует обязательной комбинированной оценки клинических и генетических данных.

**Table:** Таблица 1. Большие и малые критерии синдрома Коудена [17]

Большие критерии	Малые критерии
Макроцефалия (окружность головы >2 SD)	Расстройство аутистического спектра
Наличие родственника первого порядка с подтвержденной мутацией в гене PTEN	Умственная отсталость (IQ<75)
Рак молочной железы	Фиброзно-кистозная болезнь молочной железы
Рак щитовидной железы (не медуллярный)	Многоузловой зоб
Рак эндометрия	Миома матки
Кожно-слизистые поражения:•одна трихиллема, подтвержденная биопсией•множественные ладонно-подошвенные кератозы•мультифокальные кожные папулы на лице•макулярная пигментация головки полового члена	Расширение периваскулярного пространства по данным МРТ головного мозга
Аномалии белового вещества по данным МРТ головного мозга
Липома или липоматоз
Фибромы
Акантоз пищевода
Множественные гамартомы или ганглионевромы ЖКТ	Опухоли мочеполовой системы (особенно почечно-клеточная карцинома)

Экстраполяция взрослых критериев на детскую популяцию продемонстрирована в ретроспективном исследовании Gerdi Tuli et al., где были описаны результаты 17 историй болезни детей с мутациями в гене PTEN [9]. Средний возраст на момент постановки диагноза составлял 9,43±3,2, поражение ЩЖ присутствовало у 12 (76,5%) из 17 пациентов: у 6 — многоузловой зоб, у 5 — одноузловой зоб, у 1 — фолликулярная аденома. Макроцефалия была у 100% детей (SDS окружности головы: 3,89±1,52 см). По данным многоцентровых исследований, именно макроцефалия выявляется у всех пациентов с подтвержденной мутацией в гене PTEN и является большим критерием в постановке диагноза [9–10][19]. Gerdi Tuli et al., в своей работе также высказали важное предположение о возможной корреляции между образованиями ЩЖ и макроцефалией. При анализе историй болезни была замечена прямая зависимость, указывающая на то, что большая окружность головы (>3 SD) может быть связана с более высоким риском развития узлов щитовидной железы. Сочетание макроцефалии (SDS: 4.24) с узловыми образованиями ЩЖ в нашем случае совпадает с данным утверждением. Однако немногочисленность выборки ограничивает это предположение и требует подтверждения в более крупных когортах. Тем не менее, сочетание множественных липом, макроцефалии и узлов ЩЖ смело можно считать патогномоничными признаками, указывающими на необходимость проведения молекулярно-генетического исследования ребенку для поиска мутации в гене PTEN.

Установлено, что у детей с мутацией в гене PTEN только ДРЩЖ имеет повышенный риск развития по сравнению с другими новообразованиями. По разным источникам, частота возникновения узлов ЩЖ у детей с мутацией в гене PTEN составляет 4–12%, из них до 50% приходится на узловой зоб, потенциал злокачественности которого при данном генетическом заболевании мало изучен. Все это определяет необходимость скрининга детей с мутацией в гене PTEN на наличие узловых образований в ЩЖ, начиная с раннего возраста. Несмотря на то, что все едины в отношении необходимости скрининга на ДРЩЖ, имеются значительные расхождения по вопросам возраста его начала и методов проведения. Так, NCCN рекомендует ежегодное УЗИ ЩЖ с момента постановки диагноза [20]. В свою очередь, Американская тиреоидологическая ассоциация, во избежание гипердиагностики, советует ежегодно проводить лишь физикальные обследования (пальпацию ЩЖ), начиная с возраста постановки диагноза, с проведением УЗИ ЩЖ в случае обнаружения пальпируемых узлов, асимметрии щитовидной железы или увеличения шейных лимфатических узлов [21]. Британская группа по изучению генетики рака предлагает проводить ежегодное УЗИ ЩЖ в возрасте от 16 лет или раньше в случае наличия отягощенного по ДРЩЖ семейного анамнеза [22]. В настоящее время достигнут консенсус в отношении того, что онкологический скрининг у детей, в том числе и за опухолями с хорошим прогнозом, оправдан, если риск развития опухоли составляет 5% и более [23][24].

В зарубежной литературе есть ряд исследований, описывающих частоту развития и характер узловых образований ЩЖ у детей с мутацией в гене PTEN [8][9][10]. По данным Bubien et al., кумулятивный риск развития ДРЩЖ в детской популяции составляет 5% [25]. Ten et al., подтвердили эти цифры: у 5 из 105 пациентов (4,76%) в возрасте от 3 до 78 лет ДРЩЖ развился в возрасте до 20 лет [19]. Аналогичные результаты были получены Riegert-Johnson et al., в когорте из 211 человек ДРЩЖ развился у 4% в возрасте до 20 лет, самому молодому пациенту было 10 лет [26]. В исследовании L.A. Jonker et al., обобщены данные о частоте и особенностях ДРЩЖ при PTEN в педиатрической популяции: средний возраст развития как доброкачественный узловых образований, так и ДРЩЖ составил 12 лет (диапазон от 4 до 17 лет). Большинство случаев ДРЩЖ обнаружено в возрасте от 10 до 14 лет, наименьший возраст диагностики фолликулярной карциномы ЩЖ соответствовал 4 годам. На сегодняшний день установлен наиболее частый тип ДРЩЖ при PTEN: в 51% случаев выявляется фолликулярная карцинома ЩЖ. Столь высокая частота встречаемости фолликулярной карциномы ЩЖ, по сравнению с общепопуляционной, предполагает рассмотрение возможности использования фолликулярной карциномы ЩЖ в качестве одного из важных критериев синдрома Коудена. Имеющиеся в литературе описания клинических случаев свидетельствуют об отсутствии достоверных данных, указывающих на более агрессивное течение, повышенных рисков метастазирования или рецидивов ДРЩЖ у детей с синдромом Коудена по сравнению с детьми со спорадическим ДРЩЖ. Единственное исследование, посвященное этой теме, показало относительно низкую частоту метастазов и рецидивов у детей с мутацией в гене PTEN [27].

Объединив имеющиеся в литературе данные, с учетом всех рекомендаций, предложенных исследовательскими группами, было сформировано единое мнение о целесообразности проведения ежегодного УЗИ ЩЖ детям с мутацией в гене PTEN, начиная с 10 лет. Смысл скрининга заключается в выявлении РЩЖ на ранней стадии, предотвращении хирургических осложнений, таких как гипопаратиреоз и повреждение возвратного гортанного нерва, а также применения высоких доз радиоактивного йода.

## ЗАКЛЮЧЕНИЕ

Представленный клинический случай демонстрирует картину редкого PTEN-ассоциированного синдрома — болезни Коудена. Сочетание у ребенка рака щитовидной железы с другими злокачественными образованиями, как в нашем случае — с герминогенной опухолью яичника, указывает на возможную наследственную причину заболевания. Комбинация макроцефалии, различных кожных проявлений в виде трихеллем или папул, а также узловых образований ЩЖ в раннем возрасте говорит о необходимости проведения молекулярно-генетического исследования для поиска мутации в гене PTEN.

Начало узлообразования у ребенка в возрасте младше 7 лет и обнаружение папиллярного рака ЩЖ в 12 лет, продемонстрированные в нашем клиническом случае, еще раз, наряду с мировыми тенденциями, подтверждают необходимость ежегодного скрининга детей с синдромом Коудена на ДКЩЖ. Пациентам с подтвержденной мутацией в гене PTEN для своевременной диагностики и лечения РЩЖ рекомендуется проведение УЗИ ЩЖ один раз в год, начиная с возраста 10 лет. При необходимости хирургического лечения по поводу многоузлового зоба предпочтительным является тиреоидэктомия во избежание повторных операций в связи с частыми рецидивами узлообразования, а также неопределенным потенциалом злокачественности данных узлов. Учитывая повышенный риск развития злокачественных новообразований, присоединение других составляющих синдрома требует дальнейшего тщательного динамического наблюдения данных пациентов.

## Дополнительная информация

Источники финансирования. Работа выполнена в рамках государственного задания Минздрава РФ НИОКТР №123021000039-3.

Конфликт интересов. Авторы декларируют отсутствие явных и потенциальных конфликтов интересов, связанных с содержанием настоящей статьи.

Участие авторов. Все авторы одобрили финальную версию статьи перед публикацией, выразили согласие нести ответственность за все аспекты работы, подразумевающую надлежащее изучение и решение вопросов, связанных с точностью или добросовестностью любой части работы.

Согласие пациента. Пациент/Законный представитель пациента добровольно подписал информированное согласие на публикацию персональной медицинской информации в обезличенной форме (именно в этом журнале).
